# Coronary angiography in patients after cardiac arrest without ST-elevation myocardial infarction: A retrospective cohort analysis

**DOI:** 10.1007/s00508-021-01899-8

**Published:** 2021-06-30

**Authors:** Matthias Mueller, Daniela Dziekan, Michael Poppe, Christian Clodi, Christoph Schriefl, Martin Hofbauer, Christian Roth, Alexander Nuernberger, Michael Holzer, Christoph Weiser

**Affiliations:** 1 Department of Emergency Medicine, Medical University of Vienna, Waehringer Guertel 18–20/6D, 1090 Vienna, Austria; 2 Vienna University of Technology, Karlsplatz 13, 1040 Vienna, Austria; 3 Department of Medicine II, Division of Cardiology, Medical University of Vienna, Waehringer Guertel 18–20/6L, 1090 Vienna, Austria

**Keywords:** Resuscitation, Postresuscitation care, Percutaneous coronary intervention, NSTEMI, Catheterization

## Abstract

**Background:**

Coronary artery disease (CAD) is the most common cause of sudden cardiac arrest (SCA). Although coronary angiography (CAG) should be performed also in the absence of ST-elevation (STE) after sustained return of spontaneous circulation (ROSC), this recommendation is not well implemented in daily routine.

**Methods:**

A retrospective database analysis was conducted in a tertiary care center between January 2005 and December 2014. We included all SCA patients aged ≥ 18 years with presumed cardiac cause and sustained ROSC in the absence of STE at hospital admission. The rate and timing of CAG were defined as the primary endpoints. As secondary endpoints, the reasons pro and contra CAG were analyzed. Furthermore, we observed if the signs and symptoms used for decision making occurred more often in patients with treatable CAD.

**Results:**

We included 645 (53.6%) of the 1203 screened patients, CAG was performed in 343 (53.2%) patients with a diagnosis of occlusive CAD in 214 (62.4%) patients. Of these, 151 (71.0%) patients had occlusive CAD treated with coronary intervention, thrombus aspiration, or coronary artery bypass grafting. In an adjusted binomial logistic regression analysis, age ≥ 70 years, female sex, non-shockable rhythms, and cardiomyopathy were associated with withholding of CAG. In patients diagnosed and treated with occlusive CAD, initially shockable rhythms, previously diagnosed CAD, hypertension, and smoking were found more often.

**Conclusion:**

Although selection bias is unavoidable due to the retrospective design of this study, a high proportion of the examined patients had occlusive CAD. The criteria used for patient selection may be suboptimal.

## Introduction

Sudden cardiac arrest (SCA) is one of the leading causes of death, affecting up to 700,000 individuals in Europe every year [1]. Coronary artery disease (CAD) is the underlying problem in 75% of patients with presumed cardiac cause [2]. Therefore, immediate coronary angiography (CAG) and percutaneous coronary intervention (PCI), if appropriate, should be a substantial part of standardized postresuscitation care [3]. Based on observational studies, emergent CAG for resuscitated patients with ST-elevation (STE) myocardial infarction is recommended and leads to both increased survival and improved neurological outcomes [4].

The approach concerning CAG for patients after SCA in the absence of STE remains unclear because of conflicting data. While some studies have associated early CAG ± PCI with decreased mortality [5, 6], other studies could not show this effect [7–10]. Therefore, the European Resuscitation Council recommends providing CAG to those with the highest risk of coronary lesions [3].

However, identifying patients with the highest risk of occlusive CAD is challenging. Diagnostic tools such as electrocardiography (ECG), echocardiography, and cardiac biomarkers are of poor prognostic value in these special circumstances [11–15]. Consequentially, the European Association for Percutaneous Cardiovascular Interventions proposed to perform a short stop at the emergency department for diagnostic work-up and to detect obvious non-coronary causes; all other patients should undergo CAG as soon as possible [16]. In the recent guidelines the European Society of Cardiology encouraged the catheterization of all SCA patients without persistent ST-segment elevation when they are in cardiogenic shock. For patients who are hemodynamically stable, a delayed CAG approach should be considered [10, 17].

Despite these recommendations, CAG as a standard treatment after SCA does not seem to be implemented extensively in daily clinical routine. A systematic meta-analysis of 11 studies found that only 41.5% of the patients without STE underwent emergent CAG [18].

Therefore, we report the rate of CAG in a tertiary care center and aim to identify the factors that influence the decision pro or contra CAG diagnostics.

## Patients, material and methods

### Study setting

Vienna, the capital of Austria, had approximately 1.77 million inhabitants during the study period. Treatment for out-of-hospital cardiac arrest is provided by the municipal ambulance service, as described elsewhere [19]. We screened patients who experienced in-hospital or out-of-hospital SCA and were treated at the Department of Emergency Medicine at the Vienna General Hospital and/or by a medical emergency team. The Vienna General Hospital is a tertiary care center at the Medical University of Vienna. At the Department of Emergency Medicine, approximately 280 cardiac arrests are treated every year.

### Selection for CAG

Patient selection for CAG was performed by the attending physicians in the emergency department, intensive care units, or normal wards. During the study period, no standard operating procedures for the treatment of SCA without STE were established at our hospital. CAG ± PCI was available 24/7 although the team was on call on weekday night shifts during the study period.

### Study design and time period

We prospectively collected data of patients who received resuscitation or post-resuscitation care at our department after in-hospital and out-of-hospital cardiac arrest. Data collection, including outcome and prearrest conditions, was performed according to the Utstein criteria and stored in our local resuscitation database [20]. We screened this database for patients aged ≥ 18 years with presumed cardiac cause of arrest and sustained return of spontaneous circulation from 1 January 2005, to 31 December 2014. Trained study fellows reviewed the first ECG after admission for signs of ischemia. Patients without STE at admission were included in further analysis.

All available CAG results during the hospital stay were collected. Based on the reports of interventional cardiologists, we stratified our collective into three groups: CAG, no occlusive CAD, CAG, occlusive CAD and no CAG examination. Lesions were deemed occlusive if any kind of intervention (PCI, thrombus aspiration, coronary artery bypass grafting, CABG) was indicated.

The rate and timing of CAG were defined as the primary endpoints. As secondary endpoints, the reasons pro and contra CAG were analyzed. Furthermore, we observed whether the criteria used for decision making occurred more often in patients with treatable CAD. Favorable neurological outcome, defined as cerebral performance categories (CPC) 1–2, was reported at day 30 after return of spontaneous circulation (ROSC). As the treatment of awake patients after cardiac arrest differs from that of comatose survivors, subgroup analyses for patients with a Glasgow Coma Scale (GCS) score < 8 at admission were performed.

This study was a retrospective analysis of our resuscitation database and complied with the Declaration of Helsinki. This study was approved by the ethics committee of the Medical University of Vienna (EK 1814/2012 and 1485/2016).

### Data analysis

Categorical data were expressed as counts followed by percentages (*n* %), and differences between groups were computed using a χ^2^-test. Interval-scaled measurements were expressed as median and interquartile range (IQR). In the absence of a normal distribution as per the Kolmogorov-Smirnov test, differences between groups were determined using the Mann-Whitney U test.

We performed binary logistic regression with an inclusion model with 95% CI with a *p*-value for entry of 0.05 and *p*-value for removal of 0.1. Statistical significance was set at *p* < 0.05. All statistical analyses were performed using SPSS Statistics for MAC Version 24 and 25 (IBM, Armonk, NY, USA).

### Patient and public involvement

Patients and the public were not involved in the design and conduction of this trial.

## Results

**Fig. 1:**
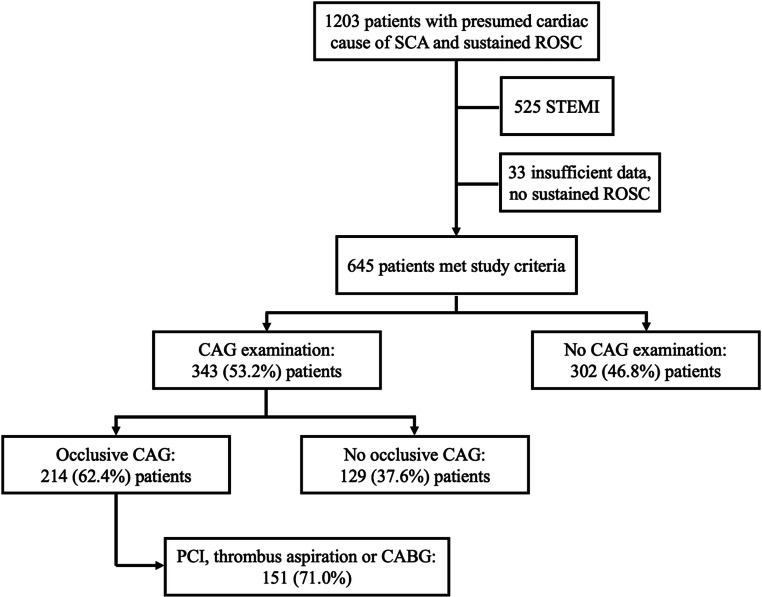
Study flowchart. *SCA* Sudden cardiac arrest, *ROSC* return of spontaneous circulation, *STEMI* ST-elevation myocardial infarction, *CAG* coronary angiography, *PCI* percutaneous coronary intervention, *CABG* coronary artery bypass grafting

During the study period, 645 (53.6%) out of 1203 screened patients with presumed cardiac causes from our resuscitation database met the inclusion criteria (*see *Fig. 1). Baseline characteristics are shown in Table 1. A CAG was performed in 343 (53.2%) patients with a subsequent diagnosis of occlusive CAD in 214 (62.4%) patients; of these, in 151 (71.0%) patients occlusive CAD was treated with PCI, thrombus aspiration or CABG (Fig. 1). PCI and/or thrombus aspiration was unsuccessful in 71 (33.2%) patients (8 patients were subsequently treated with acute CABG). After successful treatment, favorable neurological outcomes at 30 days were observed in 65.6% (*n* = 99/151). Survival rates are presented in Table 1.

**Table 1:** Baseline characteristics

	CAG (*n* = 343)	No CAG (*n* = 302)	*p*-value	CAG—occlusive CAD (*n* = 214)	CAG—no occlusive CAD (*n* = 129)	*p*-value
Age, years, median [IQR]	60 [51–68]	70 [59–79 ]	**<** **0.001**	60 [52–68]	59 [48–69]	0.253
Male sex, *n* (%)	282 (82.2)	211 (69.9)	**<** **0.001**	181 (84.6)	101 (78.3)	0.140
Body mass index, median [IQR]	27.2 [24.2–29.5]	26.6 [24.0–29.4]	0.299	27.2 [24.6–29.6]	26.9 [23.6–29.7]	0.253
Initial shockable, *n* (%)	273 (79.6)	148 (49.0)	**<** **0.001**	181 (84.6)	92 (71.3)	**0.003**
Arrest witnessed, *n* (%)	313 (91.3)	259 (85.8)	**0.028**	193 (90.2)	120 (93.0)	0.368
Bystander CPR, *n* (%)	151 (44.0)	95 (31.5)	**0.001**	83 (38.8)	68 (52.7)	**0.012**
Time to ROSC, minutes, median [IQR]	15 [6–24]	19 [7–33]	**0.007**	15 [6–23]	15 [8–25]	0.625
pH value at admission, median [IQR]	7.24 [7.14–7.31]	7.20 [7.08–7.29]	**0.006**	7.23 [7.14–7.31]	7.25 [7.16–7.32]	0.520
Lactate at admission (mmol/L), median [IQR]	5.9 [3.6–8.0]	7.2 [4.2-10.4]	**<** **0.001**	6.1 [3.7–8.2]	5.5 [3.6–7.8]	0.535
Troponin T at admission (µg/L), median [IQR]	0.048 [0.020–0.146]	0.060 [0.020–0.140]	0.422	0.057 [0.020–0.206]	0.040 [0.020–0.105]	**0.046**
proBNP at admission (pg/mL), median [IQR]	542 [140–1798]	2297 [611-6637]	**<** **0.001**	488 [128–1359]	694 [157–2157]	0.155
Creatinine (mg/dL), median [IQR]	1.27 [1.05–1.51]	1.43 [1.16–1.81]	**<** **0.001**	1.26 [1.04–1.48]	1.28 [1.07–1.54]	0.185
CPC 1 prior to CA, *n* (%)	332 (96.8)	277 (91.7)	**0.002**	208 (97.2)	124 (96.1)	0.266
GCS < 8 at admission, *n* (%)	260 (75.8)	248 (82.1)	0.122	157 (73.4)	103 (79.8)	0.139
Targeted temperature management (33 ± 1 °C), *n* (%)	246 (71.7)	205 (67.9)	0.289	144 (67.3)	102 (79.1)	**0.019**
*Cardiovascular risk factors*						
Previous diagnosed CAD, *n* (%)	118 (34.4)	141 (46.7)	**0.001**	82 (38.3)	36 (27.9)	**0.049**
Cardiomyopathy, *n* (%)	53 (15.5)	109 (36.1)	**<** **0.001**	24 (11.2)	29 (22.5)	**0.005**
Current smoking, *n* (%)	123 (35.9)	62 (20.5)	**<** **0.001**	91 (42.5)	32 (24.8)	**0.001**
Hyperlipidemia, *n* (%)	126 (36.7)	52 (17.2)	**<** **0.001**	87 (40.7)	39 (30.2)	0.052
Diabetes mellitus, *n* (%)	74 (21.6)	99 (32.8)	**0.001**	53 (24.8)	21 (16.3)	0.064
Hypertension, *n* (%)	163 (47.5)	169 (56.0)	**0.032**	111 (51.9)	52 (40.3)	**0.038**
*30–day survival*						
All patients, *n* (%)	286 (83.4)	135 (44.7)	**<** **0.001**	168 (78.5)	118 (91.5)	**0.002**
GCS < 8 at admission, *n* (%)	189 (55.1)	74 (24.5)	**<** **0.001**	104 (48.6)	85 (65.9)	**0.002**
*30–day survival (CPC1* *+* *2)*						
All patients, *n* (%)	248 (72.3)	88 (29.1)	**<** **0.001**	140 (65.4)	108 (83.7)	**<** **0.001**
GCS < 8 at admission, *n* (%)	157 (45.8)	36 (11.9)	**<** **0.001**	81 (37.9)	76 (58.9)	**<** **0.001**
Length of hospital stay in days	24 [14–39]	21 [8–51]	0.298	24 [13–45]	25 [15–36]	0.669

Baseline data—data are presented as median and [IQR] or *n* (%), *CAG* coronary angiography, *CAD* coronary artery disease, *CPR* cardiopulmonary resuscitation, *ROSC* return of spontaneous circulation, *proBNP* brain natriuretic peptide, *CPC* cerebral performance category, *CA* cardiac arrest, *GCS* Glasgow coma scale

The CAG was performed on the day of admission in 180/645 patients (27.9%). On the second day, 40/576 (6.9%, patients at risk: CAG: *n* = 330, no CAG = 246) patients underwent CAG, whereas 117/562 (20.8%, patients at risk: CAG: *n* = 327, no CAG: *n* = 235) patients were examined later during their stay (time for six patients not available) on median day 14 [8–21]. Survival with good neurological outcome for patients with GCS < 8 at admission was 31.6% for patients with CAG on the day of admission, 40% for those with CAG on day 1, and 67.5% for those with CAG later during hospital stay (*p* < 0.001 and *p* = 0.024, respectively).

Data on cardiovascular risk factors were collected and compared between the groups. Patients with pre-existing CAD, diabetes, hypertension, and cardiomyopathy were less often examined using CAG than those without these conditions (Table 1). We conducted binary logistic regression analysis to identify the factors influencing the decision to perform CAG (Table 2).

**Table 2:** Factors favoring or withholding coronary angiography

	Crude OR	95% CI	*p*-value	Adjusted OR	95% CI	*p*-value
Age ≥ 70 years	3.535	2.520–4.960	**<** **0.001**	2.512	1.692–3.729	**<** **0.001**
Male sex	0.502	0.346–0.726	**<** **0.001**	0.638	0.417–0.977	**0.039**
Arrest witnessed	0.577	0.352–0.947	**0.029**	0.621	0.344–1.123	0.115
Bystander CPR	0.584	0.422–0.806	**0.001**	0.947	0.629–1.427	0.786
Initially shockable	0.246	0.174–0.348	**<** **0.001**	0.334	0.223–0.502	**<** **0.001**
Previous diagnosed CAD	1.670	1.216–2.294	**0.002**	1.442	0.959–2.168	0.079
Cardiomyopathy	3.090	2.123–4.498	**<** **0.001**	2.274	1.484–3.484	**<** **0.001**
Current smoking	0.462	0.324–0.660	**<** **0.001**	0.771	0.509–1.169	0.221
Hyperlipidemia	0.358	0.247–0.519	**<** **0.001**	0.337	0.218–0.521	**<** **0.001**
Diabetes mellitus	1.773	1.247–2.521	**0.001**	1.186	0.777–1.810	0.430
Hypertension	1.403	1.028–1.914	**0.033**	1.031	0.707–1.505	0.873

Binary logistic regression analysis on factors favoring (odds ratio < 1) or withholding CAG (odds ratio > 1), *OR* odds ratio, *CI* Confidence interval, *CPR* cardiopulmonary resuscitation, *CAD* coronary artery disease

In a crude model, initially shockable rhythm, bystander CPR, witnessed arrest, male sex, current smoking, and hyperlipidemia were associated with high rates of CAG. After adjustment for all factors in Table 2, this effect persisted for shockable rhythm, male sex, and hyperlipidemia. In contrast, even after adjustment, age ≥ 70 years, previously diagnosed CAD, and cardiomyopathy were factors associated with lower rates of CAG.

In a binary logistic regression analysis of occlusive vs. non-occlusive CAD, initial shockable rhythm (OR 0.355; 95% CI 0.192–0.657; *p* < 0.001), current smoking (OR 0.577; 95%CI 0.346–0.962; *p* = 0.035), and hypertension (OR 0.585; 95% CI 0.358–0.956; *p* = 0.032) were the only factors that were significantly associated with a high likelihood of occlusive CAD.

After adjustment for usual cofactors of outcome, the absence of occlusive CAD and shockable initial rhythm were predictive of good neurological survival after 30 days (Table 3).

**Table 3:** Predictive factors for good neurological function (CPC 1–2) after 30 days in patients after CAG

	Adjusted OR	95% CI	*p*-value
Age, years	0.988	0.968–1.009	0.255
Arrest witnessed	1.959	0.838–4.576	0.120
Bystander CPR	0.802	0.462–1.393	0.434
Initial shockable	2.188	1.148–4.170	**0.017**
No occlusive CAD	3.037	1.695–5.442	**<** **0.001**

Binary logistic regression analysis regarding good neurological outcome (CPC 1–2) after 30 days in patients who were examined with CAG, *OR* odds ratio, *CI* confidence interval, *CPR* cardiopulmonary resuscitation

In our study cohort, 152 (23.6%) patients were women. The median age was 65.0 ([52.25–77.0]) years in women and 64.0 (53.0–73.0) years in men (*p* = 0.287). Female patients received CAG less often than male patients (57.2% vs. 40.1%, *p* < 0.001), resulting in an odds ratio of 1.99 (95%CI, 1.38–2.89) for no CAD examination in female patients. We found no statistically significant difference in PCI rates between men (41.8%) and women (41.0%, *p* = 0.902). There was no difference in the 30-day survival rate with CPC 1–2 between men (53.6%) and women (48.3%, *p* = 0.261, GCS < 8: 46.4% vs. 42.1%, *p* = 0.463). There was also no difference in the 30-day survival between men and women who underwent CAG (86.5% vs. 87.8%, *p* = 0.809) or were treated with occlusive CAD (80% vs. 84.6%, *p* = 0.584). We analyzed all baseline characteristics, risk factors, and pre-existing conditions for intersexual differences. Table 4 presents all statistically significant results, and none of the other parameters appear to be significant.

**Table 4:** Intersexual differences

	Female (*n* = 152)	Male (*n* = 493)	*p*-value
Initially shockable, *n* (%)	81 (53.3)	340 (69.0)	**<** **0.001**
Known CAD, *n* (%)	49 (32.2)	210 (42.6)	**0.023**
Cardiomyopathy, *n* (%)	49 (32.2)	113 (22.9)	**0.021**
proBNP at admission (pg/mL), median [IQR]	1774 [382–5770]	911 [214–2991]	**0.010**
Hyperlipidemia, *n* (%)	32 (21.1)	146 (29.6)	**0.039**
Smoking, *n* (%)	26 (17.1)	159 (32.3)	**<** **0.001**
CPC1 prior to CA, *n* (%)^a^	137 (90.1)	472 (96.3)	**0.003**

^a^No data available for 3 male patients. Data are presented as median and [IQR] *or n* (%), *CAD* coronary artery disease, *proBNP* brain natriuretic peptide, *CPC* cerebral performance category, *CA* cardiac arrest

## Discussion

In our retrospective analysis, we observed a CAG rate of 53.2% after the initially survived SCA. On the day of admission, CAG was performed in only about one quarter of all patients, although in those undergoing CAG, a subsequent diagnosis of occlusive CAD was frequent. We identified high age, female sex, non-shockable rhythms, and cardiomyopathy as factors associated with less CAG.

### Selection for coronary angiography

Although CAG after SCA has become an established and safe therapy over the last few decades, the current resuscitation guidelines leave the choice of patient selection and time to perform a coronary catheter examination to the physician. In our center, this decision is made by the team in charge. During the study period, young patients received CAG frequently, whereas the intention to perform CAG for older patients was low. It is known that medical professionals tend to administer less aggressive treatment to patients of advanced age [21]. We hypothesized some of the rationales behind this decision: first, there is a strong commitment to treat young, yet healthy patients, with all the possible modalities like CAG, even if the probability of CAD is relatively low. Second, comorbidities and high age are known to be independent predictors of a poor outcome [21, 22]. This knowledge may contribute to the existence of a self-fulfilling prophecy, as little effort is made in post-resuscitation care of old and chronically ill patients [23, 24].

Lemkes et al. compared early and delayed CAG in resuscitated patients without STE [10]. Although early CAG was not associated with higher survival, patients with a history of CAD and ≥ 70 years of age benefited from immediate CAG [25]. As a consequence, we arranged comparable subgroups for our regression analysis. Interestingly, high age was associated not only with delay but also with complete withholding of CAG in crude and adjusted models. As a result, patients with the highest risk of occlusive CAD did not receive CAG.

In our study population, cardiomyopathy was associated with few CAG examinations. We suppose that previous cardiac diseases are sometimes used to explain SCA as a result of fatal arrhythmias secondary to a previous myocardial scar, even if the probability of acute myocardial infarction as a trigger is high. In addition, pathological findings (e.g., wall motion abnormalities on echocardiography after ROSC) may be erroneously attributed to a pre-existing condition, thereby leading to the withholding of CAG.

Undoubtedly, all the factors mentioned above are associated with evolving difficulties during initial stabilization prior to CAG. In particular, patients with profound cardiogenic shock and insufficient response to vasoactive substances together with circumstances associated with poor neurological outcome (non-witnessed, no bystander CPR, non-shockable, long duration to ROSC) could have led to the decision to allow natural death. Of note, female sex was a predictor of non-administration of CAG. The same effect was observed in several other studies in Denmark, France, and the USA, although the underlying circumstances are not well understood [26–28].

In fact, shockable rhythms occurred less frequently, but cardiomyopathy was more often diagnosed in women than in men prior to cardiac arrest. It is possible that unmeasured confounders (e.g., few rates of reported chest pain prior to the event) also influenced the decision against CAG; however, no significant differences were found between the sexes in the percentage of patients who underwent PCI or achieved good neurological survival after CAG. The comparable PCI rates between men and women found in our study are in line with those reported in previous studies, implying the same prevalence of occlusive CAD in both sexes [27, 28]; however, the relevance of PCI in these patients remains unclear. While no difference in good neurological outcome was found in our data, Blom et al. reported lower survival rates among women than among men after cardiac arrest [29].

Perhaps CAG may be representative of an aggressive treatment bundle. Patients who were sent to CAG also received a high intensity of care concerning other modalities [6]. Consequentially, CAG could be an indication for aggressive postresuscitation care in younger patients. This selection bias also explains the remarkably high survival rates of patients without occlusive CAD. In these cases, CAG may have been performed despite lower pre-test probability (less cardiovascular risk factors, less shockable patients, lower troponin levels) just to make sure that everything possible has been done. This is also reflected by the use of targeted temperature management for almost every comatose patient in this group (99%, *n* = 102/103).

### Coronary lesions in patients without non-cardiac causes of arrest

Dumas et al. performed CAG in all patients without STE, resulting in the diagnosis of at least 1 significant lesion in 58% of the cases [5]. The recently published COACT trial found clinically significant stenosis in two thirds of the patients [10]. Both numbers are consistent with our findings. Therefore, we assume that the rate of coronary lesions would be equally high in patients who do not undergo CAG examination.

After adjustment for confounders, our analyses revealed that only shockable initial rhythm, hypertension, and smoking were associated with a high probability of occlusive CAD. While shockable initial rhythm [5, 14, 30] and smoking [5, 7] were predictive of occlusive CAD in several other studies, data regarding hypertension are conflicting. Thus, in addition to the former two factors, it is hardly possible to identify patients with occlusive CAD based on their clinical parameters.

### Limitations

This study has some important limitations. The main limitation is the lack of randomization due to the retrospective design. Therefore, we have to face a selection bias, which is inherent to all comparable studies concerning CAG after SCA. As mentioned, patients for CAG were selected at the discretion of the treating physicians. Thus, we were not able to estimate the influence of factors such as hemodynamic stability or availability of the catheterization laboratory on our findings.

Patients selected for CAG had more favorable parameters (e.g., initial shockable rhythms, younger age), which is consistent with other studies concerning this issue. It has to be considered that this could lead to falsely high survival rates [18, 31]. Another limitation is concerning the timing bias in our survival rates. If patients died early, namely in the first minutes or hours of the hospital stay and therefore never reached CAG, they are counted under “no CAG.” On the other hand, patients who did not undergo CAG until neurological recovery would naturally live longer, resulting in this bias. Thus, we suppose that the reported survival rates were overestimated.

With respect to the presented rates of patients who received CAG, we assume another limitation: data about the coronary morphology of patients who died without CAG or who did not undergo CAD examination during the hospital stay are not available. The autopsy rates after SCA in our center are too low to report substantive pathological data regarding coronary morphologies. Nevertheless, considering the comparable prevalence data from the COACT trial in which almost all patients received CAG, it is likely that CAD would be equally frequent in the patients who were not examined.

The presented findings are based on an SCA population derived from an observational and descriptive registry. Inherent to all retrospective studies, the quality of the medical records limited this study. To mitigate these problems, data collection was meticulously and rigorously validated as part of a quality improvement review. Due to changes in the database, we were not able to include patients after 2014. As the selection criteria for CAG have not changed since then, we assume that the findings remain valid to date.

## Conclusion

Although a selection bias was unavoidable due to the retrospective design of this study, a CAG rate of 53.2% with subsequent diagnosis of occlusive CAD in 62.4% of our SCA patients was found. The criteria used for patient selection may be suboptimal. For a conclusive answer about the correct timing, we urgently need more results from ongoing prospective clinical trials [32].
